# Angiogenesis in hepatocellular carcinoma: mechanisms and anti-angiogenic therapies

**DOI:** 10.20892/j.issn.2095-3941.2022.0449

**Published:** 2023-01-12

**Authors:** Changyu Yao, Shilun Wu, Jian Kong, Yiwen Sun, Yannan Bai, Ruhang Zhu, Zhuxin Li, Wenbing Sun, Lemin Zheng

**Affiliations:** 1 Department of Hepatobiliary Surgery, Beijing Chaoyang Hospital, Capital Medical University, Beijing 100043, China; 2 Department of Pathology, Peking University People’s Hospital, Peking University, Beijing 100044, China; 3 Department of Hepatobiliary Pancreatic Surgery, Shengli Clinical Medical College of Fujian Medical University, Fujian Provincial Hospital, Fuzhou 350001, China; 4 The Institute of Cardiovascular Sciences and Institute of Systems Biomedicine, School of Basic Medical Sciences, Key Laboratory of Molecular Cardiovascular Sciences of Ministry of Education, Health Sciences Center, Peking University, Beijing 100083, China; 5 Beijing Tiantan Hospital, China National Clinical Research Center of Neurological Diseases, Advanced Innovation Center for Human Brain Protection, Capital Medical University, Beijing 100050, China

**Keywords:** Angiogenesis, hepatocellular carcinoma, pro-angiogenic factors, tumor microenvironment, anti-angiogenic therapy

## Abstract

Hepatocellular carcinoma (HCC) is the fourth leading cause of cancer-associated death worldwide. Angiogenesis, the process of formation of new blood vessels, is required for cancer cells to obtain nutrients and oxygen. HCC is a typical hypervascular solid tumor with an aberrant vascular network and angiogenesis that contribute to its growth, progression, invasion, and metastasis. Current anti-angiogenic therapies target mainly tyrosine kinases, vascular endothelial growth factor receptor (VEGFR), and platelet-derived growth factor receptor (PDGFR), and are considered effective strategies for HCC, particularly advanced HCC. However, because the survival benefits conferred by these anti-angiogenic therapies are modest, new anti-angiogenic targets must be identified. Several recent studies have determined the underlying molecular mechanisms, including pro-angiogenic factors secreted by HCC cells, the tumor microenvironment, and cancer stem cells. In this review, we summarize the roles of pro-angiogenic factors; the involvement of endothelial cells, hepatic stellate cells, tumor-associated macrophages, and tumor-associated neutrophils present in the tumor microenvironment; and the regulatory influence of cancer stem cells on angiogenesis in HCC. Furthermore, we discuss some of the clinically approved anti-angiogenic therapies and potential novel therapeutic targets for angiogenesis in HCC. A better understanding of the mechanisms underlying angiogenesis may lead to the development of more optimized anti-angiogenic treatment modalities for HCC.

## Introduction

Hepatocellular carcinoma (HCC) is the seventh most common cancer and the fourth leading cause of cancer-associated mortality globally^1^. For patients with early-stage HCC, surgical resection, liver transplantation, and radiofrequency ablation are curative treatments that may provide long-term survival benefits. For patients with advanced-stage HCC, systemic treatment remains the main option, although its therapeutic benefits remain unsatisfactory. HCC is a solid tumor with a high degree of capillarization and arterialization. Therefore, angiogenesis plays important roles in the development and metastasis of HCC, particularly advanced-stage HCC.

Sprouting angiogenesis and recruitment of existing vessels by the expanding tumor mass are 2 routes through which tumor vascularity is increased. Sprouting angiogenesis, which greatly promotes tumor growth and metastasis, is the main route of formation of neovascular tumors. Angiogenesis, the formation of new blood vessels from preexisting endothelial cells (ECs), is required for cancers to obtain nutrients and oxygen, and to remove waste^2^. The oxygen and nutrients available for tumor growth become insufficient after the diameter of the lesion exceeds 1–2 mm. With rapid tumor growth, hypoxia develops within solid tumors, because of the high interstitial pressure and the distance between the tumor cells and adjacent capillaries. Angiogenesis is subsequently activated through a shift in the balance between pro-angiogenic and anti-angiogenic factors toward a pro-angiogenic mode. Pro-angiogenic factors stimulate the proliferation and migration of ECs from the vessels in the surrounding tissues. Because of its hypervascular nature, anti-angiogenic therapy is recognized as a promising therapeutic strategy for HCC. Sorafenib is the first tyrosine kinase inhibitor (TKI) approved as a standard first-line treatment for advanced HCC. Lenvatinib has been approved as a first-line treatment for advanced HCC, and regorafenib is recommended as a second-line treatment in patients with disease progression after sorafenib treatment. Cabozantinib is also used as an alternative second-line treatment^3^. Although these anti-angiogenic therapies are widely prescribed in HCC treatment, and some benefits have been noted, the survival benefits conferred by these anti-angiogenic therapies are modest^4^. In this review, we summarize current research progress in understanding the mechanisms and signaling molecules involved in angiogenesis in HCC, and discuss potential angiogenesis-targeting strategies for HCC treatment.

## Angiogenic factors

**Figure 1:**
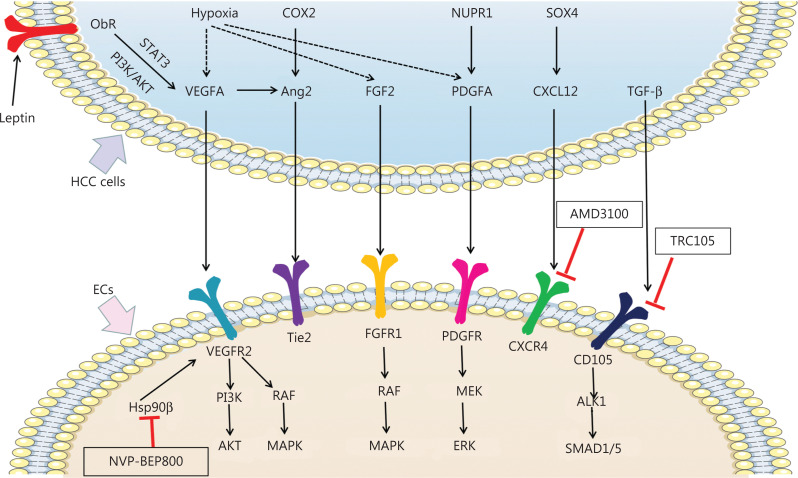
Mechanisms of pro-angiogenic factors inducing angiogenesis and new potential targets inhibiting angiogenesis in HCC. Pro-angiogenic factors secreted by HCC cells, including VEGFA, Ang2, FGF2, PDGFA, CXCL12, and TGF-β, bind the receptors expressed on ECs and promote angiogenesis in HCC. Hypoxia promotes expression of VEGFA, FGF2, and PDGFA in HCC cells. Leptin also promotes VEGFA expression by binding Ob-R in HCC cells. VEGFA binds VEGFR2 and subsequently activates the PI3K/AKT and RAF/MAPK pathways, thereby promoting angiogenesis. By interacting with FGFR1, FGF2 promotes angiogenesis in HCC by activating the RAF/MAPK pathway. PDGFA activates MEK/ERK signaling via PDGFR, thus promoting angiogenesis. TGF-β binds CD105 and activates the ALK/SMAD1/5 pathway, which promotes angiogenesis. COX2 and VEGFA induced Ang2 promotes angiogenesis by binding the Tie2 receptor. SOX4 induced CXCL12 promotes angiogenesis by binding the CXCR4 receptor. Nvp-bep800 inhibits HSP90β in ECs and subsequently attenuates angiogenesis in HCC. AMD3100 inhibits CXCR4 on ECs, thus attenuating angiogenesis in HCC. TRC105 inhibits CD105 on ECs and consequently attenuates angiogenesis in HCC.

Angiogenesis in HCC is robustly stimulated by hypoxia^5,6^. HCC consists of tumor cells, basal membrane, and surrounding stroma, and hypoxia often occurs inside tumors, owing to dense tissue structures and rapid tumor growth^7^. Hypoxia-inducible factor-1α (HIF-1α), the primary transcription factor induced by hypoxia, activates a sequence of target genes, including vascular endothelial growth factors (VEGFs), platelet-derived growth factors (PDGFs), fibroblast growth factors (FGFs), and angiopoietins, which are involved in cell survival and angiogenesis under hypoxic conditions^8,9^. In this section, we summarize the confirmed and potential mechanisms of pro-angiogenic pathways in HCC cells (**Figure 1**).

### VEGF

VEGFs, the most potent and specific regulators of ECs^10^, are critical for initiating physiological and pathological angiogenesis^11^. The VEGF family comprises 5 ligands (VEGFA, B, C, D, and E). VEGF levels reflect the aggressiveness of tumors^12^. VEGF overexpression in HCC cells enhances tumor growth and metastasis by promoting angiogenesis. Circulating plasma VEGF levels are elevated in patients with HCC and correlate with high tumor microvessel density (MVD) and poor prognosis^13^. Binding of VEGFA and VEGFB to VEGF receptor 1 (VEGFR1) leads to the formation of new vessels. The binding of VEGFA, B, C, and D to VEGFR2 stimulates the proliferation and migration of ECs, and angiogenesis^8^. The actions of VEGFC and VEGFD through VEGFR3 result in lymphangiogenesis. VEGFR2 is expressed in almost all ECs and is activated by binding of VEGFA, B, C, or D. VEGFA is the most critical ligand among these VEGFs. The binding of VEGFA/VEGFR2 leads to a phosphorylation cascade that triggers downstream cellular signaling pathways, including the PI3K/AKT and RAF/MAPK pathways, thereby resulting in ECs proliferation and migration, and the formation of branches of new blood vessels necessary for rapid tumor growth and metastasis^14,15^. Furthermore, the permeability of the newly formed vessels usually increases, thus forming areas of high interstitial pressure and severe hypoxia or necrosis, which further promote HCC progression and angiogenesis^16^.

### PDGF

PDGFs are encoded by 4 genes (PDGFA, B, C, and D) belonging to the cystine knot protein superfamily and are secreted as homodimeric proteins. PDGFs stimulate the growth and migration of glial cells, fibroblasts, and vascular smooth muscle cells^17^. PDGFs and PDGF receptors (PDGFRs) are also expressed in a variety of tumors, including HCC^18,19^. Activation of the PDGF/PDGFR signaling pathway is correlated with tumor cell proliferation and metastasis *via* modulation of multiple downstream pathways, including the PI3K/PKB and MAPK/ERK pathways^20^. In addition to stimulating cancer cell proliferation, PDGF promotes angiogenesis^21^. In HCC, elevated PDGFR-α levels correlate with MVD and poorer prognosis^8^. Olsen et al.^22^ have reported that the application of TKIs, such as imatinib, attenuates cancer cell proliferation, migration, and invasion, and affects angiogenesis by inhibiting the PDGFD/PDGFR-β axis. Li et al.^23^ have reported that the miR-325-3p–regulated CXCL17/CXCR8 axis in HCC cells regulates PDGF expression and consequently affects angiogenesis. At the molecular level, NUPR1 enhances PDGFA expression in HCC cells, and the released PDGFA facilitates angiogenesis *via* the PDGFA/MEK/ERK cascade in ECs^24^. However, the potential mechanism of the PDGF pathway as a target for angiogenesis inhibition in HCC remains unclear.

### FGF

FGFs are heparin-binding growth factors. The FGF family consists of 22 members including 18 ligands and 4 homologous factors^25^. Abnormal FGF/FGFR signaling has been reported to induce HCC; therefore, FGFs have great value as biomarkers for detecting heterogeneity in patients with HCC^26^. The FGF1, FGF2, FGF4, and FGF8 subfamilies are the most frequently investigated FGFs in the angiogenic process of HCC. Among these factors, FGF2 is the best known and researched. FGF2 is expressed in HCC cells, but is scarcely detectable in nonparenchymal cells or noncancerous liver tissue. It interacts mainly with its receptor, FGFR1, and subsequently mediates angiogenesis through the RAF/MAPK pathway^27^. FGF2 plays multiple roles in various stages of angiogenesis^28^. FGF2 not only recruits various host cells to the tumor microenvironment (TME) but also enhances VEGFA-dependent neovascularization during tumor progression—a process essential for subsequent tumor growth and metastasis^29^. FGF2 and VEGFA are associated with increased capillarization of sinusoids during angiogenesis in HCC^30^, and FGF upregulates integrin expression, which in turn alters the cellular state of ECs during angiogenesis. Wang et al.^31^ have generated the FGF2 specific monoclonal antibody GAL-F2, which not only inhibits the proliferation and migration of HCC cells, but also blocks angiogenic signals *in vivo*, thus indicating the roles of FGF2 in tumor growth and angiogenesis. These observations suggest that targeting FGF is a promising therapeutic option for anti-angiogenesis in patients with HCC.

### Endoglin

Endoglin (also known as CD105), a 180 kDa type-1 integral transmembrane glycoprotein, consists of a large extracellular domain, hydrophobic transmembrane domain, and short serine/threonine-rich cytoplasmic tail^32^. CD105 activates the activin receptor-like kinase-1 (ALK1)/SMAD1/5 or ALK4/SMAD2/3 pathways, thereby regulating cell proliferation, migration, extracellular matrix synthesis, and angiogenesis^33^. CD105 exists in both long and short isoforms. The long isoform of CD105 (L-ENG) is abundant in the human liver and has pro-angiogenic activity, whereas the short isoform of CD105 (S-ENG) is expressed mainly in senescent ECs and acts as an anti-angiogenic molecule that counters L-ENG^34^. Some studies have shown that L-ENG activates the ALK1/SMAD1/5 pathway, whereas S-ENG activates the ALK5/SMAD2/3 pathway^35^. CD105 is also a co-receptor of transforming growth factor-β (TGF-β) ligand, which is essential for fibrogenesis and angiogenesis^36^. CD105 interacts with the TGF-β family by binding TGF-β serine/threonine kinase receptors (TGF-βRs) ^37,38^. The receptor complex modulates TGF-β, SMAD1/5, and SMAD2/3 pathways^39^. CD105-induced ALK1 activation and SMAD1/5 phosphorylation are critical for enhancing the proliferation and migration of ECs, in contrast to ALK5 activation and SMAD2/3 phosphorylation-induced inhibitory effects^40^. CD105 participates in the angiogenesis of HCC^41^ and is a cell proliferation marker for vascular ECs and tumor vasculature^36^. CD105 expression is highest in well-differentiated HCC, whereas it is downregulated in poorly differentiated HCC^42^. CD105 is expressed primarily in microvessels in the tumor periphery, whereas TGF-β1 is present only within tumor hepatocytes. HCC-released TGF-β1 promotes the expression of CD105 in ECs and as a promoter of tumor angiogenesis^43^. CD105 is also significantly correlated with tumor differentiation, portal vein invasion, and lymph node metastasis^44^. Expression of CD105 in microvessels appears to be more clinically meaningful than that of CD31 or CD34^45^. In addition, compared with ECs from adjacent normal tissues, tumor-derived ECs (TECs) are resistant to drug treatment, and CD105 may play an important role in this resistance^46^. CD105+ TECs exhibit higher anti-apoptotic ability, cell motility, and stronger pro-angiogenic characteristics than CD105+ normal ECs. CD105+ TECs also demonstrate greater ability to survive in tumors. On the basis of the above data, CD105 may be a potential target for inhibiting angiogenesis and subsequent progression of HCC. TRC105 (carotuximab), a chimeric IgG1 anti-CD105 monoclonal antibody, inhibits angiogenesis and causes antibody-dependent cellular cytotoxicity and apoptosis of proliferating ECs. In an open-label single-arm phase study, Duffy et al.^47^ have combined TRC105 with sorafenib for the treatment of sorafenib-naïve patients with HCC and have found that the 2 anti-angiogenic drugs were well tolerated in combination and achieved a partial response rate of 25%. This combination treatment is currently under further evaluation in a multicenter phase II study to confirm its efficacy.

### Angiopoietin

Angiopoietin-1 (Ang1) and -2 (Ang2) are ligands of the tyrosine kinase receptor Tie2, which is expressed on ECs and promotes angiogenesis^48^. Ang1 and Ang2 are highly homologous and have similar binding affinity toward Tie2. Ang1 is a widely expressed pro-angiogenic factor in adult tissues that regulates the stabilization and maturation of newly formed vessels by enhancing endothelial cell-to-cell junctions and recruiting pericytes and smooth muscle cells^49^. In contrast, Ang2 is generally expressed during vascular remodeling processes^50^. Bupathi et al.^48^ have found that Ang2 expression increases in liver cirrhosis and is further elevated in HCC, thus indicating that the angiopoietin pathway is involved in HCC angiogenesis. Ang2, but not Ang1, significantly increases from early-stage to advanced-stage HCC, and has a high predictive power for overall survival (OS) and progression-free survival (PFS)^51^. Therefore, Ang2 may be used as a potential biomarker for both diagnosis and prognosis in HCC^52^. Generally, Ang2 antagonizes the effect of Ang1 and induces vessel regression in tumors in the absence of VEGFA. Although Ang2 attenuates vascular integrity, it stimulates EC proliferation and migration in the presence of VEGF signaling^53^. Co-overexpression of Ang2 and VEGF in HCC results in markedly greater tumor development and angiogenesis, and less intratumoral apoptosis and vessel maturation, than observed with overexpression of either Ang2 or VEGF alone. In addition, inhibition of VEGF signaling abolishes the effects of Ang2 and VEGF co-overexpression, thus indicating that Ang2 synergistically enhances VEGF-mediated HCC development and angiogenesis^54^. Wang et al.^55^ have also demonstrated that elevated expression of Ang2 and VEGFA promotes angiogenesis and stabilizes newly formed blood vessels. Several studies have demonstrated that Ang expression is not elevated in HCC cell lines; however, Ang2 released from ECs is elevated under hypoxic conditions. These observations indicate that ECs may contribute to Ang2 overexpression in the hypoxic microenvironment of HCC^56^. Further studies have shown that hypoxia-induced VEGF may upregulate the expression of Ang2 in HCC. Tanaka et al.^57^ have demonstrated that specific inhibition of cyclooxygenase-2 (COX2) attenuates Ang2 expression and angiogenesis in HCC, thereby indicating the regulatory role of COX2 on Ang2 in HCC. Therefore, Ang2 may serve as a potential therapeutic strategy for HCC, and the mechanisms underlying its regulation must be explored further.

### Leptin

Leptin is a 167-amino acid non-glycosylated protein encoded by the leptin (LEP) gene. Leptin regulates body mass by regulating energy homeostasis, and plays critical roles in immune response, reproduction, and angiogenesis^58^. During carcinogenesis, leptin stimulates cell proliferation, migration, and angiogenesis^59^. Binding of leptin to the leptin receptor (Ob-R) generates the functional unit responsible for leptin-mediated signaling. Huang et al.^60^ have reported that Ob-R enhances proliferation, migration, and invasion, and inhibits apoptosis, in HCC through regulating ERK1/2 and JAK2/STAT3 expression. Moreover, the leptin derivative OB3 abolishes leptin-induced cell proliferation by decreasing PI3K activation and proinflammatory gene expression in HCC^61^. Ribatti et al.^62^ have reported greater vascularization in poorly differentiated HCC than in other stages, and have found that leptin expression highly correlates with the degree of angiogenesis. Therefore, leptin may be involved in HCC development and angiogenesis. Leptin activates several pathways, such as JAK-STAT3, MAPK/ERK, and PI3K-AKT, thereby inducing the expression of various angiogenic factors in cancers^63^. Whether leptin might regulate angiogenesis in HCC through these pathways requires further confirmation. Several groups have developed compounds or antibodies targeting leptin/Ob-R signaling that show significant anticancer effects *in vitro* or *in vivo*, but have not been applied clinically^64,65^.

### Other pro-angiogenic factors

Recent studies have indicated that several cytokines, such as interleukins (ILs), interferons (IFNs), and TNF-α, play important roles in tumor angiogenesis. IL-1 signaling promotes angiogenesis by upregulating VEGF and other angiogenic molecules *via* the MAPK or JNK pathways^66^. IL-6, IL-18, and IL-33 also promote tumor angiogenesis by regulating the expression of angiogenic factors. Moreover, IFN-γ promotes HIF-1α expression in mesenchymal stem cells, thereby upregulating VEGF expression and promoting tumor angiogenesis^67^. Previous studies have shown that TNF-α inhibits tumor angiogenesis; however, recent studies have demonstrated that TNF-α may exert pro-angiogenic activity in tumors^68^. Tsai et al.^69^ have reported that knockout of SOX4 in HCC cells decreases the expression of CXCL12, and consequently attenuates tube formation *in vitro* and decreases angiogenesis *in vivo* in a xenograft mouse model, by targeting CXCR4 in ECs. The detailed regulatory mechanisms of tumor angiogenesis mediated by these cytokines in HCC are not well understood and deserve further investigations.

### Anti-angiogenic factors

Contrary to the effects of pro-angiogenic factors, anti-angiogenic factors such as thrombospondin-1 (TSP1), endostatin, and endorepellin antagonize angiogenesis during HCC progression.

TSP1 is a matricellular glycoprotein that modulates various cellular functions by binding extracellular proteins or cell surface receptors. It plays important roles in multiple biological processes, including angiogenesis, apoptosis, and immune regulation. TSP1 inhibits angiogenesis by directly regulating ECs proliferation, apoptosis, and migration, or by antagonizing VEGF activity^70^. Yang et al.^71^ have demonstrated that the HDAC6-let-7i-5p-TSP1 regulatory pathway suppresses neoplastic and angiogenesis in HCC. Currently, only limited studies have evaluated the role of TSP1 in HCC. Further studies are needed to identify the roles and signaling pathways of TSP1 in HCC angiogenesis.

Endostatin, generated from collagen XVIII, acts as a modifier of angiogenesis. Endostatin plays an anti-angiogenic role by inhibiting matrix metalloproteinases (MMPs), or binding α5- and αV-integrins^72^. Furthermore, endostatin mediates anti-angiogenic effects *via* activation of the downstream pathways involving the Src/Rho/Actin axis, inhibition of the FAK/Ras/MAPK/ERK signaling cascade through α5β1-integrin binding, and suppression of HIF-1α/VEGFA. Furthermore, endostatin-induced autophagy has been reported to act in concert with anti-angiogenesis and prevent HCC progression^73^. Endorepellin, the C-terminal segment of the large proteoglycan perlecan, is another anti-angiogenic factor that is involved in tumor progression and has activity similar to that of endostatin. Endorepellin binds α2β1-integrin, thus inducing disassembly of the cytoskeleton, and inhibits ECs migration. In addition, endorepellin downregulates VEGFR2 and consequently attenuates angiogenesis^74^. The specific role and mechanism of anti-angiogenic factors must be further studied.

## TME-associated cells in HCC angiogenesis

**Figure 2:**
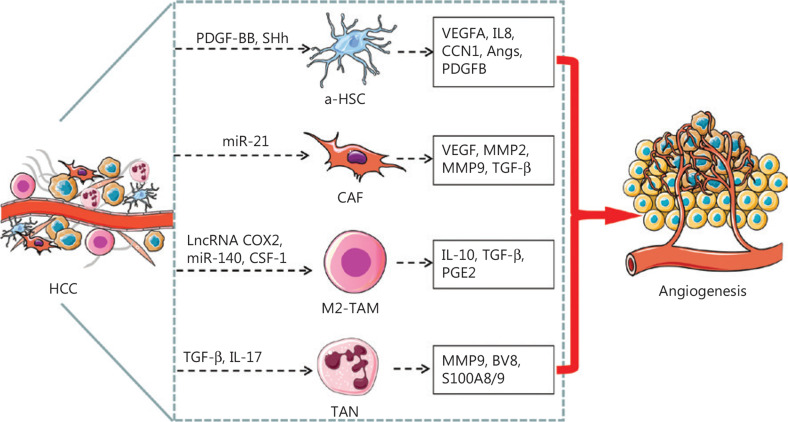
Schematic representation of TME associated cells mediating angiogenesis in HCC. The HSCs undergo a phenotypic transformation, from quiescent type to a-HSCs or to transdifferentiated CAFs, which secrete pro-angiogenic factors or cytokines that promote angiogenesis in HCC. The M2-like TAMs and TANs also secrete pro-angiogenic factors or cytokines that promote angiogenesis in HCC. PDGF-BB and SHh secreted by HCC cells activates HSC, and a-HSCs release VEGFA, IL8, CNN, Angs, and PDGFB, which in turn promote angiogenesis. Exosomal miR-21 converts HSCs to CAFs, which in turn promote angiogenesis by secreting VEGF, MMP2, MMP9 and TGF-β. LncRNA cox-2, miR-140, and CSF-1 facilitate the polarization of macrophages toward the M2 subtype, which secretes IL-10, TGF-β, and PGE2 and consequently promotes angiogenesis in HCC. TGF-β and IL-17 stimulate TANs to secrete MMP9, BV8, and S100A8/9, which promote angiogenesis in HCC.

The TME is the cellular environment in which tumors develop. Apart from tumor cells, the TME comprises various other cell types, extracellular matrix, growth factors, proteolytic enzymes, and signaling molecules^75^. The TME is closely associated with tumor growth, metastasis, angiogenesis, and drug resistance. Among the TME-associated stromal cells, hepatic stellate cells (HSCs), cancer-associated fibroblasts (CAFs), tumor-associated macrophages (TAMs), and tumor-associated neutrophils (TANs) play key roles in HCC angiogenesis (**Figure 2**). Several studies on the TME, particularly TME-associated stromal cells, such as ECs, HSCs, TAMs, and TANs, have provided valuable information for the development of anti-angiogenic therapies.

### ECs

ECs are direct target cells involved in angiogenesis. VEGFA is a specific and potent pro-angiogenic stimulator of ECs, and VEGFR2 is the most important receptor found on ECs. In addition to the hypoxia-mediated VEGFA/VEGFR, angiopoietin/tie, and CD105/TGF-β pathways, several other signaling pathways and molecules involved in angiogenesis of ECs have been identified in recent studies^76^.

Chemokine receptors on ECs are involved in angiogenesis in HCC. Treatment with the CXCR4 antagonist AMD3100 inhibits tube formation by ECs in HCC^69^. In addition, new roles of some EC molecules that may facilitate angiogenesis in HCC have been reported in recent years. Hsp90β expression is positively correlated with CD31+ MVD, and the underlying mechanism involves Hsp90β-mediated promotion of VEGFR expression *via* enhancement of their promoter activities in ECs. NVP-BEP800, a specific inhibitor of Hsp90β, significantly decreases VEGFR expression, and attenuates ECs proliferation, migration, invasion, and tubular differentiation^77^. RhoC is not only a regulator of VEGF in tumor cells but also a downstream target of VEGF in ECs, and is essential for angiogenesis. Activated RhoC is associated with increased invasion and migration of ECs through the reorganization of F-actin, which enhances the vessel network and sprout formation^78^. Dong et al.^79^ have reported that TMPRSS4 significantly promotes the expression and secretion of HB-EGF, thereby facilitating HCC angiogenesis. Treatment with specific HB-EGF inhibitor cross-reacting material 197 (CRM197) alone or in combination with sorafenib significantly inhibits angiogenesis and HCC progression.

Several studies have demonstrated that human endothelial progenitor cells (EPCs) directly mediate angiogenesis. Jamshidi-Parsian et al.^2^ have examined the intercellular crosstalk between HepG2 and human EPCs in a co-culture system mimicking some aspects of the initial tumor parenchyma and stroma interactions. Remote cell-to-cell paracrine interactions between HepG2 cells and EPCs play a critical role in the differentiation and angiogenic activity of ECs, possibly through the intercellular signaling of exosomes released in the medium by HepG2 cells. Sun et al.^80^ have transplanted green fluorescent protein (GFP)+ bone marrow cells into the bone marrow of HCC mice and found that CD31 and GFP double-positive cells are incorporated into the vessel walls, thus indicating that EPCs promote HCC angiogenesis by integrating directly into tumor vessels. Therefore, targeting ECs, even EPCs, constitutes the most direct and effective anti-angiogenic therapy for HCC.

### HSCs

HSCs, also known as liver pericytes, are located in the space of Disse. They are crucial components of the TME and promote HCC growth, metastasis, and angiogenesis^81^. In response to the TME of HCC, HSCs undergo phenotypic transformation from quiescent to activated or transdifferentiated states, thus leading to morphological, behavioral, and biochemical changes. Generally, activated HSCs (a-HSCs) express pro-angiogenic factors, including VEGFA, PDGFB, and Angs, which promote angiogenesis by binding their cognate receptors on the surfaces of ECs^81,82^. Recently, several novel mechanisms associated with a-HSC-mediated angiogenesis have been reported. A-HSCs promote hepatic vascular EC growth and microtubule formation *via* Raf signaling pathway-mediated Ang1 overexpression^83^. In addition to releasing pro-angiogenic growth factors, α-HSCs release proinflammatory chemokines that stimulate angiogenesis. Angiogenesis is active at the invading edge close to the a-HSCs, and IL-8 is enriched in the TME of HCC tissues and contributes to HCC angiogenesis *via* activation of the STAT3 signaling pathway in tumor cells^84^. Additionally, interleukin-8 (IL-8) is derived mainly from a-HSCs, but not from hepatoma cells, in HCC. Cellular communication network factor-1 (CCN1) expressed by a-HSCs promotes the growth of HCC xenografts *in vivo*, affects the function of a-HSCs, and regulates the formation of the xenograft microenvironment, such as fibrogenesis and angiogenesis^85^. Mußbach et al.^86^ have found that proteinase-activated receptor-2 (PAR2) expressed by LX-2 cells promotes tumor growth and angiogenesis in HCC xenografts in mice. Inhibition of PAR2 attenuates TGF-β1 induced Smad2/3 activation, and blocks the secretion of pro-angiogenic and pro-mitotic factors and proteinases; therefore, HSC-derived PAR2 plays a key role in promoting HCC progression by mediating tumor cell migration and angiogenesis.

The crosstalk between HCC cells and HSCs also affects angiogenesis. PDGF-BB secreted by HCC cells under hypoxic conditions stimulates the proliferation and accumulation of HSCs in the TME and consequently leads to the release of VEGFA, which in turn promotes HCC angiogenesis^87^. Huh-7-derived sonic hedgehog (SHh) activates hedgehog signaling in HSCs, thus leading to increased secretion of angiogenic factors, which in turn promote angiogenesis in HCC^88^. On the basis of these data, Li et al.^89^ have developed a novel GA-sHA- doxorubicin polymer for targeted co-delivery of capsaicin and doxorubicin to tumor cells and HSCs, which effectively inhibits SaP-HSCs-HCC axis-induced tumor metastasis and angiogenesis.

### CAFs

CAFs are major components of the tumor stroma in HCC and are closely associated with tumor initiation, angiogenesis, progression, and chemoresistance^90^. CAFs not only interact directly with HCC cells and other stromal cells in a paracrine manner, but also remodel the TME components and consequently create a microenvironment conducive to tumor cell invasion and metastasis, and HCC progression. Activated CAFs exhibit increased secretory capacity and secrete cytokines, such as TGF-β, insulin-like growth factor, hepatocyte growth factor, and interleukin-6 (IL-6), thus promoting proliferation, metastasis, and angiogenesis in HCC^91^.

Current research has indicated that CAFs are derived from multiple cell types including normal resident fibroblasts, HSCs, mesenchymal stem cells, epithelial cells, and ECs^92^. One study has established that 85% of CAFs transdifferentiate from HSCs in HCC^93^. HCC cell-derived exosomal miR-21 converts HSCs to CAFs by inhibiting PTEN and activating PDK1/AKT^94^. Transdifferentiated CAFs further promote angiogenesis by secreting angiogenic cytokines, including VEGF, MMP2, MMP9, and TGF-β. Huang et al.^95^ have shown that CAFs promote HCC angiogenesis by secreting VEGF and consequently the enhancer of Zeste homolog-2 (EZH2)/vasohibin 1 (VASH1) pathway in ECs. A recent study has also shown that CAFs overexpress placental growth factor (PLGF), which is specifically associated with angiogenic markers, including CD31, CD34, and CD105^96^. Thus, CAF-derived PLGF may be an effective therapeutic target for arresting CAF-regulated tumor angiogenesis in HCC. In addition to their supportive role in angiogenesis, some specific CAF subsets play opposite roles in tumor blood vessels. The various origins of CAFs may contribute to the heterogeneity of their functions in angiogenesis. For instance, a subset of CAFs derived from portal fibroblasts secrete prolargin, which binds and antagonizes pro-angiogenic growth factors, including TGF-β1, FGF1, FGF2, and hepatocyte growth factor, and subsequently inhibits angiogenesis in HCC^97^.

### TAMs

The phenotypes of macrophages change dynamically depending on the duration and degree of inflammation, fibrosis, and cancer. Macrophages are classified into M1 and M2 subtypes according to their functional heterogeneity. M1 macrophages constitute the proinflammatory and anti-tumorigenic subtypes, which express TNF-α, IL-1β, IL12, CCL2, iNOS, and ROS, all of which are important for HCC initiation. In contrast, M2 macrophages represent the anti-inflammatory and pro-tumorigenic phenotype that express arginase-1 and CD206, which creates an immunosuppressive environment favorable for HCC development.

Oliveira et al.^98^ have found that the macrophage influx number did not change in the lesions of a high fat diet fed zebrafish HCC model; however, the number of TNFα-positive macrophages and the level of angiogenesis were higher in the HCC + high fat diet group than the HCC control group. This result suggests that macrophages, and specifically macrophage polarization, play key roles in the early progression and angiogenesis of non-alcoholic fatty liver disease/non-alcoholic steatohepatitis-associated HCC. In addition, metformin treatment rescues macrophage polarization, angiogenesis, and tumor progression. Suppression of the long noncoding RNA (lncRNA) COX2 attenuates the ability of M1 macrophages to inhibit HCC cell proliferation, invasion, migration, and angiogenesis, while strengthening the ability of M2 macrophages to promote the proliferation of HCC cells and angiogenesis^99^. HCC-derived HOMER3-AS1 promotes HCC progression by increasing macrophage recruitment and M2-like polarization. Investigation of the mechanism has revealed that HOMER3-AS1 promotes the HCC malignant phenotype by activating HOMER3/Wnt/β-catenin signaling, and facilitates macrophage infiltration and M2-like polarization by upregulating CSF-1^100^. Hou et al.^101^ have reported that MALAT1 in HCC promotes the production of VEGFA and facilitates the polarization of macrophages toward the M2 subset *via* miR-140, thus accelerating HCC metastasis and angiogenesis. Recent studies have suggested that the M2-like TAMs enhance the production of IL-10, TGF-β, and PGE2, and consequently promote tumor angiogenesis^102^. In addition, TAMs may inhibit anti-tumor T cells in the initial stage of HCC development and contribute to early T cell exhaustion, thus enhancing tumor progression and angiogenesis.

### TANs

TANs have emerged as important components of the TME that play important roles in angiogenesis and tumor proliferation^103^. During hepatocarcinogenesis, neutrophil density significantly increases in HCC^98^. TANs facilitate the recruitment and polarization of macrophages and regulatory T cells, thereby enhancing angiogenesis in HCC^104^. Yan et al.^105^ have found that suppressing neutrophil differentiation inhibits hepatocarcinogenesis; therefore, TANs are pro-tumorigenic during the initiation of hepatocarcinogenesis, in agreement with previous data suggesting that TANs promote tumor proliferation and angiogenesis. TANs sustain tumor angiogenesis through the release of the pro-angiogenic factors MMP9, BV8, and S100 proteins S100A8/9. TAN-derived MMP9 enhances the liberation and activation of VEGFA and consequent angiogenesis, whereas BV8 acts as a mitogen for ECs^106^.

### Other TME-associated cells

Other TME-associated cells, such as T-regulated cells, dendritic cells, and tumor-associated adipose cells, also secrete pro-angiogenic factors, such as VEGFA, MMP9, IL10, TGF-β, and leptin, and consequently promote cancer angiogenesis^66,107^. Cytotoxic T lymphocytes (CTLs) play anti-angiogenic roles in tumor angiogenesis. Recruitment of both endogenous and exogenous CTLs specifically targets tumor vasculature^108^. CD8+ CTLs efficiently secrete IFN-γ, thus inhibiting tumor growth and angiogenesis in hepatocellular carcinoma^109^. However, research on the role of these adaptive immunes cells in HCC angiogenesis is lacking, and further investigation is required.

## Cancer stem cells (CSCs) in HCC angiogenesis

CSCs are a rare population of tumor-initiating cells that drive tumor initiation and growth as well as tumor metastasis, recurrence, and resistance to chemo- or radiotherapy. Some cell surface proteins, including CD133, EpCAM, CD90, CD44, CD24, CD13, and ICAM-1, have been identified as CSC biomarkers^110^. Among them, the stemness properties of CD133-, CD90-, and EpCAM-enriched cells has been characterized in primary HCC^111,112^. Therapies targeting CSCs significantly affect cancer progression.

Emerging evidence indicates that CSCs are directly associated with angiogenesis, promotion of tumor growth, and metastasis in HCC^110^. High expression levels of HSC/hematopoietic progenitor cell biomarkers are positively correlated with tumor angiogenesis and poor prognosis in HCC^113^. These results imply that CSCs may affect angiogenesis in HCC, and thus cooperatively promote tumor development, and may have negative effects on patient outcomes in HCC. More recently, several studies have elucidated the effects of CSCs on angiogenesis in HCC. Tang et al.^114^ have determined that CD133+ CSCs have an enhanced ability to secrete IL-8 and markedly induce tumor angiogenesis. One possible underlying mechanism is that neurotensin enhances IL-8 and CXCL1 expression, and consequently activates the MAPK signaling cascade in CD133+ CSCs, thus increasing the ability of CSCs to induce angiogenesis. Conigliaro et al.^115^ have reported that exosomes containing lncRNA H19 released by CSC-like CD90+ cells, but not by parental hepatoma cells, modulate the angiogenic phenotype of ECs and cell-to-cell adhesion, thus promoting angiogenesis in HCC. Dickkopf-1 (DKK-1) is highly expressed in CSCs in HCC, and DKK-1 supplementation activates angiogenesis in vascular ECs^116^. Cheng et al.^117^ have reported that Src/FAK phosphorylation activates CSCs, thereby inducing cell migration and angiogenesis. The authors have further documented that combination treatment with sorafenib and dasatinib suppresses cell migration and angiogenesis by targeting Src/FAK phosphorylation, thus decreasing cell-to-cell contact, cancer stem cell activation, and VEGF secretion, and suppressing angiogenesis. These studies have demonstrated that CSCs play critical roles in angiogenesis in HCC and therefore contribute to recurrence and resistance to angiogenic treatment in patients with HCC. In addition to general angiogenesis, vasculogenic mimicry (VM) is a new tumor vascular paradigm in which aggressive cancer cells form vessel-like networks that provide adequate blood supply for tumor growth and metastasis. VM is also an important mechanism contributing to the failure of currently available angiogenesis inhibitors for HCC^118^. Recent studies have highlighted that CSCs are closely associated with VM formation^119,120^. The lncRNA n339260 promotes VM by inducing CSC in HCC^119^. Frizzled2 (FZD2) promotes CD44+ stem-like properties and a VM phenotype in HCC *via* the Hippo signaling pathway, which may serve as a potential therapeutic target in HCC^120^.

**Figure 3:**
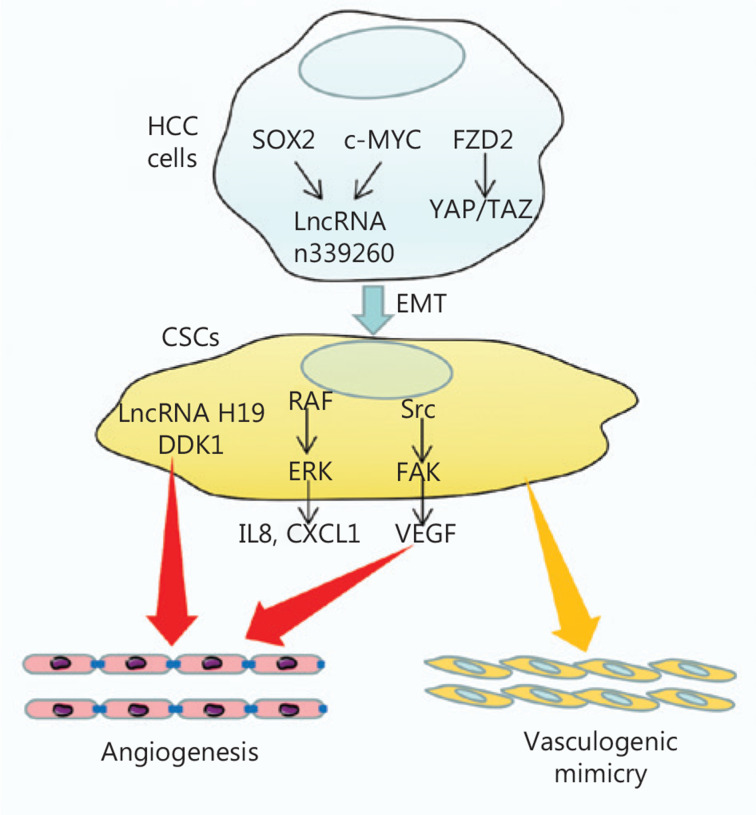
Mechanisms of CSCs in HCC angiogenesis. SOX2 and c-MYC regulate lncRNA n339260, thus promoting VM through the induction of a CSC-like phenotype in HCC. FZD2 also promotes VM through the induction of the CSC-like phenotype through the YAP/TAZ pathway. Meanwhile, CSCs promote angiogenesis by secreting IL8 and CXCL1 through the RAF/ERK pathway, or secreting VEGF through the Src/FAK pathway. CSCs also secrete lncRNA H19 and DDK1, thus promoting angiogenesis in HCC.

Briefly, CSCs secrete pro-angiogenic factors and exosomes that promote angiogenesis. In contrast, CSCs contribute to resistance to anti-angiogenic therapy in HCC *via* VM formation. On the basis of these results, an effective anti-angiogenic therapy should target CSCs for HCC treatment (**Figure 3**).

## Anti-angiogenic therapies in HCC angiogenesis

Anti-angiogenic therapy is a promising strategy for the treatment of HCC (**Table 1**). Sorafenib is the first TKI approved as a first-line treatment for advanced HCC^121^. Sorafenib blocks receptors involved in oncogenesis and angiogenesis, including VEGFR2, PDGFR, Raf-1, c-Kit, FLT3, and RET. In the Sorafenib HCC Assessment Randomized Protocol (SHARP) trial, the sorafenib group showed a longer OS than the placebo group (10.7 months *vs.* 7.9 months; HR 0.69; *P* < 0.001)^122^. Another phase III trial has indicated similar results demonstrating a significant improvement in OS (6.5 months *vs.* 4.2 months; HR 0.68; *P* = 0.014) and time to progression (2.8 months *vs.* 1.4 months; HR 0.57; *P* = 0.0005) in patients treated with sorafenib rather than placebo^123^. Analysis of the 2 phase III trials testing sorafenib has revealed a consistent survival benefit across all clinical subgroups. Common treatment-associated adverse events of sorafenib include diarrhea (39%), fatigue (22%), hand-foot skin reaction (21%), rash or desquamation (16%), and anorexia (14%). Lenvatinib^124^, an oral inhibitor of VEGFR, FGFR, PDGFR, RET, and c-Kit, has been approved as a first-line treatment for HCC, on the basis of an open-label, phase III, multicenter, non-inferiority trial. This clinical trial showed that lenvatinib was non-inferior to sorafenib in terms of OS (13.6 months *vs.* 12.3 months; HR 0.92)^125^. Furthermore, lenvatinib, compared with sorafenib, also elicited a significant improvement in PFS (7.4 months *vs.* 3.7 months; HR 0.66; *P* < 0.0001) and objective response rate (ORR) (40.6% *vs.* 12.4%; OR 5.01; *P* < 0.0001). In a subgroup analysis, patients with baseline serum AFP levels >200 ng/mL had greater benefit from lenvatinib than sorafenib (HR 0.78, 95% CI 0.63–0.98). The most common adverse events in lenvatinib treatment included hypertension (42%), diarrhea (39%), decreased appetite (34%), weight loss (31%), and fatigue (30%). Donafenib, a novel multi-kinase inhibitor with efficacy and safety superior to those of sorafenib, is recommended as a first-line agent for HCC in China^126^_._ An open-label, randomized, parallel-controlled, multicenter phase II–III trial has indicated that donafenib exhibited superior OS outcomes to sorafenib (12.1 months *vs.* 10.3 months; HR 0.831; *P* = 0.0245), although the PFS was 3.7 *vs.* 3.6 months (*P* = 0.057). Moreover, drug-associated grade ≥ 3 adverse events occurred in fewer patients receiving donafenib than sorafenib (38% *vs.* 50%; *P* = 0.0018)^127^. The superior OS benefit with donafenib was observed across most subgroups, and a statistically significant improvement in OS was achieved in some subgroups. Hand-foot skin reactions (50%), diarrhea (30%), decreased platelet count (28%), hypertension (26%), and elevated AST (23%) were the most common adverse events associated with donafenib treatment.

**Table 1:** Summary of anti-angiogenic agents for HCC

Anti-angiogenic agents	Application	Targets	Trial name (identifier)	Clinical results	Common adverse events
Sorafenib	First-line	VEGFR2, PDGFR, Raf-1, c-Kit, FLT3, RET	SHARP (NCT00105443)	OS: 10.7 *vs.* 7.9 months (HR 0.69; 95% CI: 0.55–0.87, *P* < 0.001)	Diarrhea (39%), fatigue (22%), hand–foot skin reaction (21%), rash or desquamation (16%), anorexia (14%)
				Time to radiologic progression: 5.5 *vs.* 2.8 months	
				(HR 0.58; 95% CI: 0.45–0.74, *P* < 0.001)	
				ORR: 2% *vs.* 1%	
Lenvatinib	First-line	VEGFR, FGFR, PDGFR, RET, c-Kit	REFLECT (NCT01761266)	OS: 13.6 *vs.* 12.3 months (HR 0.92; 95% CI: 0.79–1.06)	Hypertension (42%), diarrhea (39%), decreased appetite (34%), decreased weight (31%), fatigue (30%)
				PFS: 7.4 *vs.* 3.7 months (HR 0.66; 95% CI: 0.57–0.77, *P* < 0.0001)	
				ORR: 40.6% *vs.* 12.4%	
Donafenib	First-line (China)	VEGFR, PDGFR, Raf	ZGDH3 (NCT02645981)	OS: 12.1 *vs.* 10.3 months (HR 0.831; 95% CI: 0.699–0.988, *P* = 0.0245)	Hand–foot skin reaction (50%), diarrhea (30%), decreased platelet count (28%), hypertension (26%), elevated AST (23%)
				PFS: 3.7 *vs.* 3.6 months (HR 0.909; 95% CI: 0.763–1.082, *P* = 0.0570)	
				ORR: 4.6% *vs.* 2.7%	
Atezolizumab plus bevacizumab	First-line	PD-L1, VEGF	IMbrave150 NCT03434379	Survival rates at 12 months: 67.2% *vs.* 54.6%	Hypertension (29.8%), fatigue (20.4%), proteinuria (20.1%), aspartate aminotransferase increase (19.5%), pruritus (19.5%)
				PFS: 6.8 *vs.* 4.3 months (HR 0.59; 95% CI: 0.47–0.76, *P* < 0.001)	
				ORR: 33.2% *vs.* 13.3%	
Regorafenib	Second-line	VEGFR, PDGFR, FGFR1, RET, c-Kit	RESORCE (NCT01774344)	OS: 10.6 *vs.* 7.8 months (HR 0.63; 95% CI: 0.50–0.79, *P* < 0.0001)	Hand–foot skin reaction (53%), diarrhea (41%), fatigue (40%), hypertension (31%), anorexia (31%)
				PFS: 3.1 *vs.* 1.5 months (HR 0.46; 95% CI: 0.37–0.56, *P* < 0.0001)	
				ORR: 11% *vs.* 4%	
Cabozantinib	Second-line	VEGFR, HGFR, c-Kit, RET, FLT-3, Tie2, Axl	CELESTIAL (NCT01908426)	OS: 10.2 *vs.* 8.0 months (HR 0.76; 95% CI: 0.63–0.92, *P* = 0.005)	Diarrhea (54%), decreased appetite (48%), palmar–plantar erythrodysesthesia (46%), fatigue (45%), nausea (31%)
				PFS: 5.2 *vs.* 1.9 months (HR 0.44; 95% CI: 0.36–0.52, *P* < 0.001)	
				ORR: 4% *vs.* <1%	
Ramucirumab	Second-line	VEGFR2	REACH-2 (NCT02435433)	OS: 8.5 *vs.* 7.3 months (HR 0.71; 95% CI: 0.531–0.949, *P* = 0.0199)	Fatigue (24%), peripheral oedema (24%), decreased appetite (22%), bleeding or hemorrhage events (19%), proteinuria (18%)
				PFS: 2.8 *vs.* 1.6 months (HR 0.452; 95% CI: 0.339–0.603, *P* < 0.0001)	
				ORR: 5% *vs.* 1%	
Apatinib	Second-line	VEGFR2	AHELP (NCT02329860)	OS: 8.7 *vs.* 6.8 months (HR 0.785; 95% CI: 0.617–0.998, *P* = 0.048)	Hand–foot skin reaction (56%), hypertension (48%), platelet count decreased (46%), proteinuria (44%), aspartate aminotransferase increased (38%)
				PFS: 4.5 *vs.* 1.9 months (HR 0.471; 95% CI: 0.369–0.610, *P* < 0.0001)	
				ORR: 11% *vs.* 2%	

Regorafenib is recommended for HCC progression after sorafenib treatment^128^. Regorafenib—a multi-kinase inhibitor that targets VEGFR, c-Kit, RET, PDGFR, and FGFR1—provides a survival benefit regardless of the rate of disease progression during prior sorafenib treatment or since the last sorafenib administration^129^. Regorafenib treatment resulted in a longer OS than did the placebo (10.6 months *vs.* 7.8 months; HR 0.63; *P* < 0.0001)^128^. The PFS also improved significantly (3.1 months *vs.* 1.5 months; HR 0.46; *P* < 0.0001). The improvement in OS and PFS with regorafenib was maintained in all subgroups. Hand-foot skin reactions (53%), diarrhea (41%), fatigue (40%), hypertension (31%), and anorexia (31%) were more common during treatment with regorafenib. Cabozantinib is also used as an alternative second-line treatment for HCC. Cabozantinib is a TKI that inhibits VEGFR, hepatocyte growth factor receptor (HGFR), c-Kit, RET, FLT-3, Tie2, and Axl. Both the median OS (10.2 months *vs.* 8.0 months; HR 0.76; *P* = 0.005) and PFS (5.2 months *vs.* 1.9 months; HR 0.44; *P* < 0.001) were longer in the cabozantinib arm than the placebo arm. Furthermore, the ORR in the cabozantinib arm was higher than that in the placebo arm (4% *vs.* less than 1%)^130^. All subgroup analyses favored treatment with cabozantinib in PFS and OS, except in patients with HCV or patients of Asian descent. Further analyses are necessary to help understand these differences. Diarrhea (54%), decreased appetite (48%), palmar-plantar erythrodysesthesia (46%), fatigue (45%), and nausea (31%) were common adverse events associated with cabozantinib.

In addition to the above-mentioned multi-target inhibitors, ramucirumab, a single-target inhibitor, is also recommended as a second-line targeted agent for HCC. Ramucirumab, a human recombinant IgG1 monoclonal antibody targeting VEGF2, has been associated with better survival benefits in patients with HCC with AFP ≥ 400 ng/mL or higher^131,132^. Patients in the ramucirumab arm, compared with the placebo arm, showed a significantly prolonged OS (8.5 months *vs.* 7.3 months; HR 0.710; *P* = 0.0199) and PFS (2.8 months *vs.* 1.6 months; HR 0.452; *P* < 0.0001). All subgroup analyses favored treatment with ramucirumab in OS and PFS, except in female patients, which included only 16 patients in the placebo group. However, no statistical difference in ORR was observed between the ramucirumab and placebo arms^131^. Fatigue (24%), peripheral edema (24%), decreased appetite (22%), bleeding or hemorrhage events (19%), and proteinuria (18%) were the adverse events associated with ramucirumab treatment. Notably, apatinib, a novel VEGFR2 TKI, has attracted considerable attention and shown promising anti-tumor effects in sorafenib-resistant HCC^133^. In the AHELP trial, a randomized, double-blind, placebo-controlled, phase III trial, the OS was significantly longer in the apatinib group than the placebo group (8.7 months *vs.* 6.8 months; HR 0.785; *P* = 0.048). The PFS was also significantly longer in the apatinib than the placebo group (4.5 months *vs.* 1.9 months; HR 0.471; *P* < 0.0001). The superior OS benefit with apatinib was observed across most subgroups, except in patients older than 65 years of age. The most common treatment-associated adverse events included hand-foot syndrome (56%), hypertension (48%), decreased platelet count (46%), proteinuria (44%), and increased aspartate aminotransferase (38%)^134^.

Furthermore, a combination of targeted therapy with immune checkpoint inhibitors (ICI) achieved superior efficacy to monotherapy in HCC^135^. Atezolizumab plus bevacizumab was approved as a first-line therapy for patients with unresectable or metastatic HCC, on the basis of the phase III IMbrave150 trial in 2020^136^. Atezolizumab is a human monoclonal IgG1 antibody that targets PD-L1 and blocks its interactions with PD-1 and B7.1. Bevacizumab is a monoclonal anti-VEGF antibody. In this phase III study, the OS at 12 months in the atezolizumab plus bevacizumab arm was longer than that in the sorafenib arm (67.2% *vs.* 54.6%). The median PFS in atezolizumab plus bevacizumab arm was also longer than that in the sorafenib arm (6.8 months *vs.* 4.3 months; HR 0.59; *P* < 0.001)^136^. The benefit of OS and PFS with atezolizumab–bevacizumab, compared with sorafenib, was generally consistent across the clinically relevant subgroups analyzed. The most common treatment-associated adverse events included hypertension (29.8%), fatigue (20.4%), proteinuria (20.1%), increased aspartate aminotransferase (19.5%), and pruritus (19.5%). The combination of ICI with anti-angiogenesis agents appears likely to change the treatment paradigm for HCC. The possible underlying mechanism is that the combination of ICI with anti-angiogenesis agents may activate more anti-tumor immune cells and decrease the immune inhibitory components to a greater extent than ICI alone^137^.

Despite the impressive results of anti-angiogenic therapies in HCC, several challenges remain. First, current anti-angiogenic clinical trials induce only a modest improvement in OS, measurable in just several months. Given that HCC has multiple pathways for recruiting vessels, blocking one target alone may have incomplete effects on tumor angiogenesis, because the tumor cells may switch from one mechanism to another^132,138^. However, the use of anti-angiogenic molecules may lead to a hypoxic TME, which alters the cell phenotype and enhances tumor invasiveness, whereas the tumor-infiltrating cells, including TAMs, TANs, and TAFs, may decrease the response to anti-angiogenic therapies^139^. Drug resistance is also a major cause of failure of anti-angiogenic therapies. The underlying mechanisms may be tumor heterogeneity and clonal evolution during treatment. In addition, drug-associated adverse events may lead to dose reduction, interruption, or discontinuation, thereby decreasing the therapeutic effect. Consequently, overcoming these challenges and exploring more efficient strategies with low-toxicity are directions for future research.

## Conclusion and perspectives

HCC is the seventh most common malignancy. Patients are usually diagnosed with HCC in advanced stages and consequently have unfavorable clinical prognosis. HCCs are hypervascular tumors that tend to develop an aberrant vascular network and angiogenesis. Accumulating evidence indicates that angiogenesis is closely correlated with HCC growth, progression, invasion, and metastasis. In this review, we provided an overview of the current research on signaling pathways that regulate angiogenesis *via* pro-angiogenic factors secreted by HCC and CSCs, and other TME-associated cells (**Table 2**).

**Table 2:** Molecules and mechanisms of angiogenesis in HCC

Cell types	Molecules or factors	Mechanism	Phenotype	Reference
HCC cells	VEGFA	VEGFA/VEGFR2/PI3K/AKT	Angiogenesis	^15^
		VEGFA/VEGFR2/RAF/MAPK		
	PDGFA	PDGFA/PDGFR/MEK/ERK		^24^
	FGF2	FGF2/FGFR1/RAF/MAPK		^27^
	TGFβ	TGFβ/CD105/ALK1/SMAD1/5		^43^
	Ang2	COX2/Ang2/Tie2		^57^
	CXCL12	SOX4/CXCL12/CXCR4		^69^
	TSP1	HDAC6-let-7i-5p-TSP1	Anti-angiogenesis	^71^
HSCs	Ang1	Raf/Ang1	Angiogenesis	^83^
	IL8	IL8/STAT3		^84^
	CCN1	CCN1/Cyr61		^85^
	PAR2	PAR2/TGFβ/Smad2/3		^86^
CAFs	VEGF	VEGF/EZH2/VASH1	Angiogenesis	^95^
	PLGF	–		^96^
TAMs	IL10, TGFβ, PGE2	–	Angiogenesis	^102^
TANs	MMP9, BV8, S100A	–	Angiogenesis	^106^
CTLs	IFN-γ	–	Anti-angiogenesis	^109^
CSCs	IL-8, CXCL1	RAF/ERK/IL-8, CXCL1	Angiogenesis	^114^
	lncRNA H19	–		^115^
	DKK-1	–		^116^
	VEGF	Src/FAK/VEGF		^117^
	LncRNA n339260	SOX2, c-MYV/LncRNA n339260	Vasculogenic mimicry	^119^
	FZD2	FZD2/YAP/TAZ		^120^

To develop anti-angiogenic therapies with better efficacy, studies have focused on mechanisms that may influence angiogenesis in HCC. ECs are the most relevant cells directly associated with angiogenesis, and all pro-angiogenic factors and other molecules ultimately act on ECs. Consequently, understanding the mechanisms of ECs during angiogenesis is crucial for developing new anti-angiogenic therapies. HSCs undergo phenotypic changes resulting in the expression of pro-angiogenic factors, including VEGFA, PDGFB, and Ang, thereby enhancing angiogenesis. Additionally, HSCs can transdifferentiate into CAFs and consequently promote angiogenesis. TAMs and TANs are immune cells that exist in tumors and mediate tumor progression. The role of CSCs in angiogenesis in HCC has been elucidated in recent years. CSCs not only release pro-angiogenic factors and exosomes that enhance angiogenesis, but also facilitate VM formation, thus enhancing resistance to anti-angiogenic therapy. Current understanding of the TME is limited. Therefore, an in-depth investigation of TME associated cells may provide new therapeutic strategies for the treatment of HCC angiogenesis.

Although anti-angiogenic therapies for HCC have achieved some impressive results, several challenges remain, including a modest improvement in OS and drug resistance. Alternative therapeutic strategies, including the use of multiple anti-angiogenic compounds or anti-angiogenic drugs in combination with other treatment therapies, may improve therapeutic effects and overcome resistance to anti-angiogenic therapy. For instance, TKI and ICI combination treatment has achieved positive results in several clinical trials and has been approved as a breakthrough first-line therapy for advanced HCC. In addition, the efficacy of combined therapies should be monitored during disease progression to optimize and counteract the development of further resistance. The integration of multidisciplinary therapies for angiogenesis in HCC and development of personalized treatments will contribute to the improvement of HCC treatment.

Multiple mechanisms are involved in angiogenesis in HCC. Further studies are needed to explore the mechanisms of angiogenesis in diverse compositions of HCC to identify new targets for anti-angiogenic therapies or novel combination therapies aimed at improving outcomes for patients with HCC.
